# kegg_pull: a software package for the RESTful access and pulling from the Kyoto Encyclopedia of Gene and Genomes

**DOI:** 10.1186/s12859-023-05208-0

**Published:** 2023-03-04

**Authors:** Erik Huckvale, Hunter N. B. Moseley

**Affiliations:** 1 Markey Cancer Center, University of Kentucky, Lexington, KY 40536 USA; 2 Department of Molecular and Cellular Biochemistry, University of Kentucky, Lexington, KY 40536 USA; 3 Institute for Biomedical Informatics, University of Kentucky, Lexington, KY 40536 USA

**Keywords:** KEGG, REST, Application programming interface, Python programming language, Command line interface

## Abstract

**Background:**

The Kyoto Encyclopedia of Genes and Genomes (KEGG) provides organized genomic, biomolecular, and metabolic information and knowledge that is reasonably current and highly useful for a wide range of analyses and modeling. KEGG follows the principles of data stewardship to be findable, accessible, interoperable, and reusable (FAIR) by providing RESTful access to their database entries via their web-accessible KEGG API. However, the overall FAIRness of KEGG is often limited by the library and software package support available in a given programming language. While R library support for KEGG is fairly strong, Python library support has been lacking. Moreover, there is no software that provides extensive command line level support for KEGG access and utilization.

**Results:**

We present kegg_pull, a package implemented in the Python programming language that provides better KEGG access and utilization functionality than previous libraries and software packages. Not only does kegg_pull include an application programming interface (API) for Python programming, it also provides a command line interface (CLI) that enables utilization of KEGG for a wide range of shell scripting and data analysis pipeline use-cases. As kegg_pull’s name implies, both the API and CLI provide versatile options for pulling (downloading and saving) an arbitrary (user defined) number of database entries from the KEGG API. Moreover, this functionality is implemented to efficiently utilize multiple central processing unit cores as demonstrated in several performance tests. Many options are provided to optimize fault-tolerant performance across a single or multiple processes, with recommendations provided based on extensive testing and practical network considerations.

**Conclusions:**

The new kegg_pull package enables new flexible KEGG retrieval use cases not available in previous software packages. The most notable new feature that kegg_pull provides is its ability to robustly pull an arbitrary number of KEGG entries with a single API method or CLI command, including pulling an entire KEGG database. We provide recommendations to users for the most effective use of kegg_pull according to their network and computational circumstances.

**Supplementary Information:**

The online version contains supplementary material available at 10.1186/s12859-023-05208-0.

## Background

The Kyoto Encyclopedia of Genes and Genomes (KEGG) [1–3] is a collection of databases containing organized biomolecular and metabolic data (information) for over 3000 species with sequenced genomes. A primary component of each KEGG database is a KEGG entry, a relational table record that represents and describes a specific chemical, biochemical, or biological entity (e.g. a chemical compound, a biochemical reaction or pathway, an enzyme, a gene, a species etc.). Each KEGG entry is uniquely identified with a KEGG ID. The KEGG databases are updated regularly and made publicly available via the KEGG website [4]. However, the website is designed for manual access through a web browser. For more automated access, KEGG provides a Representational State Transfer (REST) web application programming interface (web API). A REST web API is a predominant software architecture for making uniform interactions between software components via the World Wide Web. These interactions typically occur as requests in the form of a uniform resource locator (URL) provided through the http protocol, with a “GET” http request fetching data from a web server [5]. The KEGG REST web API (KEGG API) [1] provides a set of operations for accessing most of the organized data in KEGG as described on the KEGG API web page: https://www.kegg.jp/kegg/rest/keggapi.html. In particular, the KEGG API enables researchers to retrieve KEGG data, especially KEGG entries, for use in their own analyses. Operations to obtain KEGG entry IDs include the “list” and “find” operations, the output of these operations returning meta data which needs to be parsed out if only the entry IDs themselves are desired. And the “get” operation provides KEGG entries themselves given their corresponding IDs.

Users can make requests to REST web APIs by providing the correct URL to a variety of web accessing software, for example a web browser, library packages like the Python requests module [6], and even command line tools like cURL [7]. However, construction of these URLs is somewhat cumbersome, requiring specific URL templates for a specific REST web API with some URL construction expertise, which is even limiting for some bioinformaticians, let alone biologists with limited computational skills. Library packages do exist both in R [8] and Python [9] for accessing most of the KEGG API. However, to our knowledge, none of these packages provide a command line interface (CLI) for researchers who prefer to use the command line or to write shell scripts. Also missing is a package that provides a variety of other use cases, for example obtaining KEGG entry IDs alone with the metadata already parsed out or downloading an arbitrary number of entries in a single command. Therefore, we introduce a new Python package kegg_pull, which meets the above use cases and more. We have implemented kegg_pull to a rigorous industrial standard, which includes both unit and integration tests. The kegg_pull package is installable through the Python Package Index (https://pypi.org/project/kegg-pull/).

We created kegg_pull to promote the FAIR (Findable, Accessible, Interoperable, and Reusable) guiding principles of data stewardship [10] with respect to KEGG. While KEGG is primarily responsible for implementing FAIR, kegg_pull improves on the accessibility, interoperability, and reusability of the KEGG API. The kegg_pull package improves the accessibility by making the utilities of the KEGG API accessible to Python programmers, including those that may have limited knowledge of web development. Additionally, it makes these utilities accessible to command line users either for shell scripting or for executing one-time commands without needing to write any script at all. Interoperability is improved by making the output from the KEGG API available in a form suitable for other contexts, such as Python objects in a python script, files in the file system, and console output that can be piped into another command on the command line. This necessarily allows KEGG data to be used in shell scripts. The improved interoperability enables the output to be transferred downstream within a complex workflow or to be used by a workflow manager. Finally, the kegg_pull package improves reusability by making KEGG data more easily reused by researchers in a variety of Python-based data analyses and command line-based data analysis pipelines.

### Implementation

The kegg_pull package provides several useful CLI and API features for interacting with the KEGG API. This includes wrapper methods/commands for all the REST API operations, pulling lists of KEGG entry IDs, and pulling an arbitrary number of KEGG entries that are automatically separated and saved into individual files, all with a single function call or command line execution. Also, the package provides robust multiprocessing pull functionality specifically designed to mitigate blacklisting from the KEGG API triggered by a rapid series of REST operations.

**Fig. 1:**
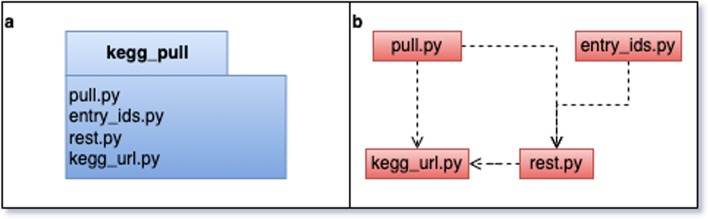
**a** UML package diagram. **b** Submodule dependencies

The kegg_pull API is implemented in four submodules (Fig. 1): pull, entry_ids, rest, and kegg_url. See the Additional file 1 for additional implementation details of these submodules. The kegg_pull CLI reuses this API to provide a higher level of functionality, conveniently accessible from the command line without needing to write Python scripts. If more flexibility is necessary, however, researchers with programming expertise can use the kegg_pull API in their own Python scripts and programs.

The kegg_url submodule constructs URL objects for accessing the KEGG REST API (See Additional file 1: Fig. S1). The kegg_rest submodule uses these URLs to provide wrapper methods over each of the KEGG REST API operations via its KEGGrest class (See Additional file 1: Fig. S2). A user-created Python program could use the kegg_url submodule to construct the URLs and, if more control over the URLs is needed, pass them into a Python library such as requests. However, the benefits of using the wrapper methods of the KEGGrest class include:

1. Abstracting the URL strings so less knowledge of web development is needed and using the requests library under the hood automatically.
2. Allowing the caller to specify the number of tries to make a request in case initial requests fail or time out.
3. Allowing the user to specify how long requests should wait for a response before being marked as timed out.
4. Allowing the caller to specify the sleep time in between requests that time out or are blacklisted to give the KEGG web server time to return to an accessible state. Blacklisting is when the KEGG web server temporarily blocks further requests when it deems too many have been made, necessitating waiting until the blacklisting is repealed.
5. Returning a KEGGresponse object (see Additional file 1: Fig. S2) which contains the information from a response generated from a request to the KEGG API, including both a text body and binary body if applicable, the URL constructed for the request, and the status (i.e. SUCCESS, FAILED, or TIMEOUT).

The KEGGrest wrapper methods provide the exact output from the KEGG REST API, which is the desired outcome in some use cases. However, in many cases, additional processing is desired. That is why we provide additional submodules including the entry_ids submodule, which uses the rest submodule to provide methods for getting lists of KEGG entry IDs (See Additional file 1: Fig. S3). A user-created Python program could use the KEGGrest class directly to get the entry IDs from its relevant methods. However, the benefits of using the methods in the entry_ids submodule include:

1. The response body comes as a string that contains metadata on top of the entry IDs. The entry_ids module will additionally parse the string to return a list containing only the entry IDs themselves.
2. The entry_ids submodule also contains a method for loading a list of entry IDs from a file if the user already has the entry IDs they’d like to retrieve in their local file system.

**Fig. 2:**
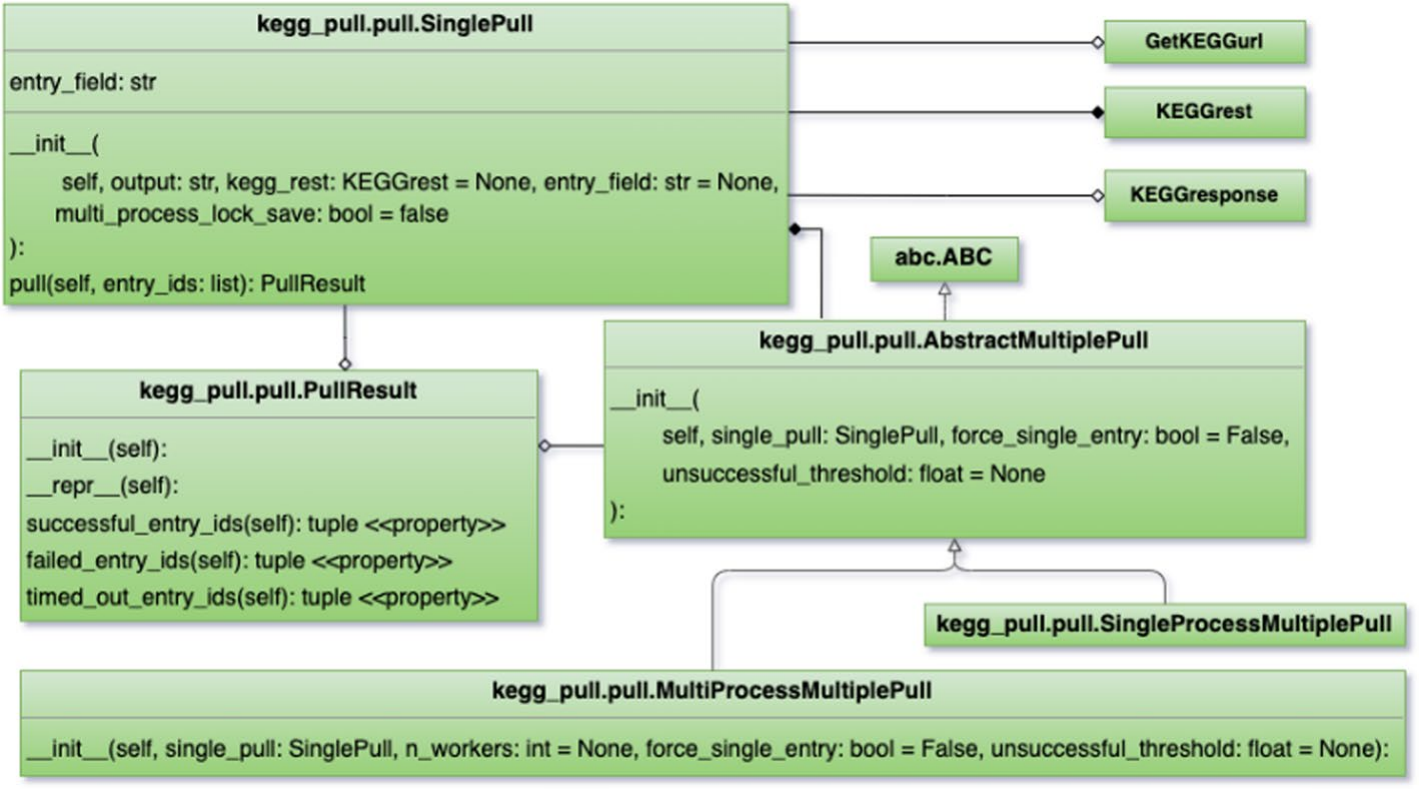
Class diagram of the pull.py module

As with the entry_ids submodule, the pull submodule also provides very helpful post-processing on top of the raw output from the KEGG REST API. The pull submodule provides classes that use the “get” method of the KEGGrest class to pull KEGG entries into individual files in the user’s local file system, a very common use case of KEGG users. This includes the ability to pull KEGG entries in their default format or to pull specific entry fields from them (e.g. the mol file of a compound entry, the JSON file of a KEGG Brite entry, the image file detailing a compound’s molecular structure, the nucleotide sequence of a gene etc.). The classes in the pull submodule include the SinglePull class which has a "pull" method (see Fig. 2) that makes just one request to the KEGG REST API to pull one or more entries. One could use the KEGGrest class’s “get” method directly to obtain the entry or entries from the response body. However, the benefits of using the pull method of the SinglePull class are:

1. The string or bytes response is automatically saved to the file system with the entry ID as the file name and the entry field as the file extension, the “.txt” extension used if no entry field is specified (entry is saved in the default format).
2. If the response body is binary, the file is automatically saved in binary format.
3. If multiple entry IDs are provided, the entries are automatically split by their respective delimiter in the response body and saved separately in individual files, sparing the user from needing to perform the same empirical experiments we did during software development to determine what the delimiters are in the first place and additionally sparing them from needing to write their own parser functions.
4. If multiple entries are requested and the initial request fails or not all requested entries were returned, each entry is requested one at a time (instead of them all being requested in a single response) to maximize the number of successful entries pulled.
5. The user can specify to save the output file in a regular directory or a zip archive file. If the provided directory name ends in “.zip”, the file is automatically saved in a ZIP archive of that name. If either the provided directory or provided ZIP archive doesn’t already exist, one will be automatically created.
6. A PullResult object (Fig. 2) is returned specifying by their ID which of the entries requested were successfully pulled, which entries failed to be pulled, and which entries timed out.
7. The SinglePull class, with the multi_process_lock_save parameter set to True in the constructor, will block other processes from executing the file saving code block, making files saved to the same ZIP archive multi-process safe.

Since the SinglePull class makes only one request to the KEGG REST API, its “pull” method can only pull as many entries as allowed by KEGG for a single request. The pull submodule provides additional classes, namely SingleProcessMultiplePull and MultiProcessMultiplePull, which also have a “pull” method (see Fig. 2). These classes are not limited to the number of entries pulled but rather they can pull an arbitrary number of entries in a single function call. A user-created Python program could have its own loop which calls a SinglePull object’s pull method multiple times, if desired. However, using the MultiplePull classes has the following benefits:

1. The MultiplePull classes already have a loop built into their “pull” method which makes as many requests to the KEGG REST API as necessary in order to pull all of the entries requested by the user. This spares the user from needing to implement their own loop.
2. The “pull” method optimizes the requests by splitting the provided list of entry IDs into a list of lists to take advantage of KEGG’s ability to provide multiple entries per request. Each individual request is limited to a maximum amount of entries as described above but since the list of lists contain lists no longer than this maximum amount, the pulling is optimized without exceeding that limit on a given request. The user is spared from implementing this complex functionality in order to optimize the pulling.
3. While the SinglePull class returns a PullResult object for an individual request, the MultiplePull classes provide a comprehensive PullResult detailing the merged results of all requests made.
4. The MultiplePull classes display a progress bar in the console.
5. They additionally provide the ability to halt the program if too many of the requests fail or time out. The user can also specify a failure rate threshold for automatic halting.
6. While both the SingleProcessMultiplePull and the MultiProcessMultiplePull classes will pull all of the requested entries, the MultiProcessMultiplePull class enables pulling entries across multiple processes to pull more entries in less time when running on a system with multiple cores. The user can specify the number of processes to use, the default being the number of cores available.
7. Multiprocessing is safe in the case of saving files to a regular directory since each file is written entirely within its own process rather than multiple processes writing to that same file. However, it is not safe when writing files to a ZIP archive. While the processes are writing different files to this ZIP archive, the ZIP archive itself is technically a single file which multiple processes write to. Having multiple processes writing to a single ZIP archive creates a race condition, which will corrupt the ZIP archive when multiple processes open and write to it at the same time. The MultiProcessMultiplePull takes precautions to make writing to ZIP archives safe even in a multi-processing context (as long as its SinglePull member has its multi_process_lock_save parameter set to True; see Fig. 2), sparing the user from concern over these low-level details.

**Fig. 3:**
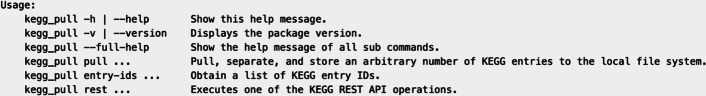
Top level command line usage of kegg_pull

The top-level command line interface usage description in Fig. 3 shows that kegg_pull has 3 subcommands, namely rest, entry-ids, and pull. These subcommands reuse the rest, entry_ids, and pull submodules and are analogous to the entities within them. However, the command line interface provides additional functionality. For the rest and entry-ids subcommands, the user can choose whether to print the output to the console or save it in a file. Similar to the pull methods in the API, the user can choose to save within a regular directory or within a ZIP archive. Using the pull subcommand on the command line makes the progress bar visible in the console and saves the information contained within the PullResult to a file. Not only are the successful, failed, and timed out entries specified (by their ID) in this file, but other useful information about the pull is saved as well, including the time it took to pull all the requested entries, the success percent or percent of entries that succeeded out of the total number of requested entries, and the amount of each entry ID category, i.e. the number of entries that succeeded to be pulled, the number that failed, etc. If the user instructs kegg_pull to abort upon too many entries not succeeding, a file detailing the results of the aborted pull is created.

The kegg_pull CLI enables shell scripting in addition to python scripting depending on a user’s needs. This allows for complete reproducibility of data analysis pipelines. However, providing this functionality in a command line shell enables one-time data retrieval for prototyping prior to or alongside development of the pipelines. Since researchers often perform a high amount of experimentation and investigation before generating the final results, one-time data retrieval can provide immediate data for quick information or experiments. In such cases, writing a script to do so is unnecessary and premature. While KEGG itself provides an interactive browser that fills this need to an extent, the kegg_pull CLI provides the following additional benefits beyond the KEGG browser:

1. Those comfortable with the command line may find it more efficient to type in a single command and readily get the data they need. Even for merely viewing data a single time (printing to the console), entering a single CLI command can be quicker than opening another window and navigating to the particular web page they need which may require navigating to one page after another.
2. Again, even if the user merely wants to view data a single time, the KEGG browser can only display a single KEGG entry at a time via its graphic user interface. The kegg_pull CLI can print multiple entries at time. One could type in the exact KEGG REST API URL into their browser’s search bar, but this is hardly more effective than passing the URL into a command line program like cURL or Postman where the user has to manually construct the URL. The kegg_pull CLI constructs these URLs for the user with an intuitive interface.
3. While the above benefits are for merely viewing data, if the user wants to actually save data, not only do they need to navigate to the KEGG browser web page they need, but they also need to download the data. The kegg_pull CLI can perform that additional step by simply specifying an—output parameter.
4. If the user is working on a remote machine, they’d have to both download the data from the browser to their local machine and then transfer it to their remote machine. The kegg_pull CLI can download it directly to the remote machine.
5. If the user wants to download multiple entries from the KEGG browser, they’d have to repeat the above steps for each entry. The kegg_pull CLI can pull an arbitrary number of entries in a single “pull” subcommand. The “entry-ids” subcommand additionally parses entry-ids to be displayed in the console or saved in a file.

More details on the kegg_pull API and CLI is available in the online package documentation: https://moseleybioinformaticslab.github.io/kegg_pull/.

## Results

### Sleep time performance

The kegg_pull CLI enables the user to pull all the entries in a specified KEGG database with a single command. We discovered that the time it takes to accomplish this varies based on the—sleep-time option (the time to wait in between timed out requests and blacklisted requests). This option also affects the success percentage, the percentage of entries that succeed rather than fail. When we performed the execution time experiments (Tables 1 and 2), we found that none of the requests timed out, so the results most likely reflect the percentage of successfully pulled entries as compared to those that were blacklisted for all three tries. Since each request only tried 3 times, waiting for 0 s in between tries would not give enough time to wait for the KEGG web server to repeal the blacklisting. This is most likely why we see an increase in the success percentage as the sleep time increases. After reaching 100%, increasing the sleep time unsurprisingly no longer affects the success percentage. Our results in Table 1 also show a negligible increase in pull time after increasing sleep time past reaching 100% success in the case of the KO database. In the case of the larger VG database shown in Table 2, we actually see a continued decrease in pull time after reaching 100% success. See the Additional file 1 for the single process version of this experiment (ko database only). From that table, we see that even a sleep time of 0.0 can result in 100% success when pulling in a single process.

**Table 1:** Pull success percentage and time spent pulling by sleep time—KO database (25,439 entries)

Sleep time (seconds)	0.0	0.5	1.0	2.0	3.0	5.0	10.0
Percent success	94.68	94.89	96.78	99.78	100.0	100.0	100.0
Pull time (minutes)	12.99	16.03	14.69	10.82	8.51	8.44	8.7

Number of minutes spent attempting to pull all the entries in the KO KEGG database. Percent success is the percentage of the entries in the KO database that were successfully pulled while the others failed. Difference in pull time and percent success varies by the—sleep-time option on the kegg_pull CLI. All other options remained the same, including the use of multiprocessing. Values were collected on a 12 core (hyperthreaded) machine using 12 processes

**Table 2:** Pull success percentage and time spent pulling by sleep time—VG database (595,443 entries)

Sleep TIME (seconds)	0.0	0.5	1.0	2.0	3.0	5.0	10.0
Percent success	82.04	86.98	94.6	98.21	100.0	100.0	100.0
Pull time (minutes)	555.51	663.17	416.35	366.88	215.66	204.96	194.71

Same as Table 1 except for the VG KEGG database

In the case of the Brite KEGG database, 20 entries consistently failed despite increases in sleep time. We can conclude that these 20 entries are simply unavailable rather than resulting from indeterministic blacklisting. The “list” KEGG REST operation provides the entry IDs of an entire KEGG database. After attempting to pull the entries corresponding to the Brite IDs returned by the “list” operation, not all of the entries were available as tabulated in Table 3. See the Additional file 1 for the list of the Brite entry IDs that fail.

**Table 3:** Failed entries in the brite database regardless of sleep time

Database name	Number Of successful entries	Number Of failed entries	Total entries	Success rate (percent)
Brite	118	20	138	85.51

The number of entries in the Brite database that are successfully pulled compared to the number that are not available. The failed entries fail despite increasing sleep time to wait for a blacklist to be overturned. This means they are truly unavailable, despite being output from the list operation for the corresponding database, and they did not merely fail due to a temporary blacklisting during run time

### Multiprocessing performance

When making multiple requests to the KEGG REST API to pull an arbitrary number of entries, a kegg_pull user can specify in both the API and CLI to use one process or multi-processing. As illustrated in Tables 4 and 5, we see that the pull time for whole KEGG databases can be dramatically reduced when using multi-processing.

**Table 4:** Multi-process pull time versus single process pull time (minutes) into a regular directory

Database name	Multi-process pull time	Single process pull time	Number Of entries
Pathway	0.1	1.05	558
Compound	6.4	73.62	19,004
KO	8.32	74.0	25,458

The amount of time to pull and save all the entries of a given database on a single process (one core) compared to pulling across multiple processes (multiple cores). The above values result from running kegg_pull on a 12 hyper-threaded core machine using 12 processes for multiprocessing and one process for single-processing. The sleep time and all other options for each were also constant. Files were saved in a regular directory

**Table 5:** Multi-process pull time versus single process pull time (minutes) into a ZIP archive

Database name	Multi-process pull time	Single process pull time	Number Of entries
Pathway	0.42	1.13	558
Compound	38.09	73.62	19,004
KO	66.74	138.44	25,458

Same as Table 4 except files were saved in a ZIP archive

We see that pulling KEGG entries into a ZIP archive significantly increases pull time as compared to pulling into a regular directory. However, multi-process pulling into a ZIP archive is still substantially faster than single process pulling into a ZIP archive, despite process locking the code block that accesses the ZIP file, which is required to prevent corrupting the ZIP archive file.

### Multiple entry request performance

Table 6 demonstrates the substantial increase in pull efficiency from the KEGG API’s ability to request multiple entries within a single response body. The success percentage can also decrease slightly when only pulling one entry per request, necessitating increased sleep time.

**Table 6:** Pull Time (minutes) and percent success with one entry at a time versus ten entries at a time and different sleep times

Database name	One entry at a time				Ten entries at a time			
	Sleep time 5 S		Sleep time 20 S		Sleep time 5 S		Sleep time 20 S	
	Percent success	Pull time	Percent success	Pull time	Percent success	Pull time	Percent success	Pull time
Module	98.69	1.34	100.0	1.59	100.0	0.06	100.0	0.07
Pathway	98.75	1.6	100.0	1.67	100.0	0.11	100.0	0.09
Compound	99.24	63.62	100.0	61.36	100.0	6.77	100.0	7.21
KO	99.39	83.91	100.0	90.4	100.0	8.42	100.0	9.22

The amount of time (minutes) to pull all the entries from a given database and the success percentage when pulling one entry at a time (with the—force-single-entry flag set) compared to pulling ten entries (maximum allowed by KEGG) per request. Each of these are compared to a lower sleep time vs. a higher sleep time. Results were collected on a 12 (hyperthreaded) core machine on 12 processes with all other options consistent

When pulling entries from KEGG, there is a maximum number of entries that can be pulled in a single request due to KEGG API response limitations. While the entries of all the KEGG databases, except for Brite, support requesting this maximum amount, that is not necessarily the case when a user desires to pull particular fields from the entries. For example, a user might want to pull an amino acid sequence from a gene entry or a mol file from a compound entry. Some entry fields allow this maximum number while others do not. While this is not currently specified in the KEGG REST documentation, we empirically discovered which entry fields allow this and which only allow a single entry to be pulled at a time. With this information shown in Table 7, we implemented kegg_pull such that it will pull only one entry at a time if the user wants an entry field that does not support multiple for a single request. Likewise, if the user specifies to pull all the entries from the Brite database, kegg_pull will only pull one entry at a time in that case as well. That convenience for the Brite database, however, is only available in the CLI when the database name is specified. When pulling Brite entries in all other cases, there isn’t a way to tell which database the entries are coming from, necessitating the force_single_entry parameter for the API and the—force-single-entry option for the CLI. Even if the user neglects to set this parameter/option, however, kegg_pull is robust enough to retry on each requested entry individually if not all of the requested entries are pulled initially. Forcing a single entry at a time is for efficiency rather than successful pulls.

**Table 7:** Entry fields that allow multiple entries to be pulled versus those that only allow one per request

Entry field	Can pull multiple entries in one request
aaseq	✓
ntseq	✓
mol	✓
kcf	✓
image	×
conf	×
kgml	×
json	×

One can pull up to 10 entries with a single request to the KEGG REST API. Pulling more entries per request can dramatically reduce pull time and increase the success percentage (see Table 6). However, this option is not available for all pulls. While this is not specified in the KEGG REST API documentation, nor do their requests fail if we request ten entries when only one is supported for a given entry field/database (they simply return the first entry in the request and exclude the other requested entries without any notification), we empirically determined which entry fields allow multiple entries per request and those that don’t. One can specify the field of an entry to pull rather than the standard “flat file format” (not available for Brite entries). While the flat file format pulls can pull multiple entries per request, some of the field entries can while others can’t. In addition to what’s displayed in this table, entries from the Brite database cannot be pulled more than one at a time per request, as Brite entries are not available in flat file format

While the KEGG REST API documentation explicitly states a 10-entry limit for the "get" operation, it does not specify such a limit for any other operations that accept a sequence of parameters (e.g. the keywords for the "find" operation, the entry IDs for the "ddi" operation, etc.). Such operations include "find", "conv", "link" and "ddi". We experimented with increasing the number of parameters with such operations and found no evidence of limits to the parameters themselves as with the "get" operation. What we did discover, however, was there is a limit to the number of characters in the request URL itself. We noticed that requests with a URL length of above 4000 characters consistently failed with a non-200 status code and we suspect that KEGG is using an older Apache webserver (or configuration) as part of the KEGG REST API implementation, which often limits http(s) requests to 2^12 = 4096 bytes including the headers with the LimitRequestLine parameter in the server configuration file. As a result of these experiments, we added a check to the AbstractKEGGurl class (and necessarily those classes that extend it) which ensures the URL is no more than 4000 characters long, otherwise it raises an exception informing the user that the URL is too long. We recommend that kegg_pull users, who find themselves in this edge case, break up their overly long URL into multiple requests.

### API and CLI examples

Since the CLI builds off of the API, a kegg_pull user can write API code that’s analogous to corresponding CLI commands. We say analogous rather than synonymous because the CLI can do more than the analogous API commands (e.g. saving the output to a file or printing to standard output rather than merely returning a Python object). When a user chooses to use the API over the CLI, they sacrifice potential convenience for higher control, if needed. Table 8 has examples of prominent API usage followed by their analogous CLI commands in Table 9.

**Table 8:** API examples

Action	Examples
Pull Entries with a single request	import kegg_pull.pull as p single_pull = p.SinglePull(output = 'kegg-entries/') single_pull.pull(entry_ids = ['cpd:C00001', 'cpd:C00002'])
Pull Entries with multiple requests	import kegg_pull.pull as p import kegg_pull.entry_ids as ei single_pull = p.SinglePull( output = 'kegg-entries.zip', entry_field = 'mol' ) multi_pull = p.MultiProcessMultiplePull( single_pull = single_pull, n_workers = 4 ) entry_ids: list = ei.from_file(file_path = 'entry-ids.txt') multi_pull.pull(entry_ids = entry_ids)
Pull entry IDs	import kegg_pull.entry_ids as ei ei.from_database(database_name = 'hsa')
REST operation	import kegg_pull.rest as r kegg_rest = r.KEGGrest() kegg_response: r.KEGGresponse = kegg_rest.molecular_find( database_name = 'drug', exact_mass = (200, 220) )
URL creation	import kegg_pull.kegg_url as ku conv_url = ku.DatabaseConvKEGGurl( kegg_database_name = 'hsa', outside_database_name = 'ncbi-geneid' )

Example method calls from the API, executable in a python script or python console. The above lines of code are analogous to the corresponding terminal commands in Table 9. While the API requires more lines of code than the CLI, it allows users to use kegg_pull functionality in their own python scripts. There is also no CLI commands for URL creation but those using the API can use this functionality if they just want KEGG REST URLs. Finally, there is no analogous distinction between SinglePull and AbstractMultiplePull in the CLI but rather there is only a pull command

**Table 9:** CLI examples

Action	Examples
Pull Entries	cat entry-ids.txt | kegg_pull pull entry-ids - –multi-process –n-workers = 4 –output = kegg-entries.zip –entry-field = mol
Pull entry IDs	kegg_pull entry-ids database hsa
REST operation	kegg_pull rest find drug –exact-mass = 200 –exact-mass = 220

Example terminal commands from the CLI. The above terminal commands are analogous to the corresponding lines of code in Table 8. Notice that the analogous CLI commands can do in one line what took the API several lines of code. Also note that there is no distinction between a multiple pull or single pull in the CLI. Under the hood, the CLI uses a concrete class of AbstractMultiplePull for all pulls since it can handle any number of entries, including only one entry

## Discussion

Other projects were also considered for the comparison done in Table 10. These projects include KEGG-Crawler with the home page of https://github.com/mentatpsi/KEGG-Crawler, KEGGtools with the home page of https://github.com/FlyPythons/KEGGTools, and django-rest-kegg with the home page of https://pypi.org/project/django-rest-kegg/. They were considered for comparison since they contain code for accessing the KEGG API and downloading KEGG data. However, they give the user no control over which KEGG entries to download but rather choose for the user which entries/data to download, suggesting they are for a more specific purpose than our general purpose kegg_pull package and the other projects compared in Table 10. Additionally, some of these projects are not installable packages but can only be cloned as git repositories, making importing entities into user projects or running scripts on the command line more cumbersome. So we did not deem them appropriate for comparison to kegg_pull.

**Table 10:** Package information about kegg_pull and related packages

Package name	Home page	Python version	Available both On GitHub And PyPi	Last updated
kegg_pull	https://pypi.org/project/kegg-pull/	≥ 3.8	Yes	2022
KEGGutils	https://pypi.org/project/KEGGutils/	≥ 3.8	Yes	2022
biopython (Bio.KEGG.REST)	https://pypi.org/project/biopython/ (https://biopython.org/docs/latest/api/Bio.KEGG.REST.html)	≥ 3.6	Yes	2021
keggrest	https://pypi.org/project/keggrest/	2.7	Yes	2013

The new kegg_pull python package makes available the features of the popular R package known as KEGGREST [11] in that it provides an API that wraps the KEGG REST interface, making it easier to make REST requests and doing so in a way that can be automated within user-created Python scripts. While other Python packages (Table 10) [12–14] have replicated some of the functionality of KEGGREST, kegg_pull provides a more functional API than all of these packages (Table 11), a complete CLI with a superset of the API functionality (Table 11), and is written to an industrial software engineering standard. Perhaps the most significant feature introduced with kegg_pull is its ability to make multiple requests such that it can pull an arbitrary number of entries with a single command, including the ability to do so in a multi-processing manner. This ability, however, is not without caveats. If a user requests an especially high number of entries in a single call, such as tens of thousands or more, the frequency of blacklisting increases with the number of requested entries. While we cannot prevent blacklisting, the sleep time can be optimized to maximize the success percentage while keeping the overall pull time low. The best sleep time to choose evidently must be higher when requesting a higher number of entries. While there isn’t a mechanism to predict what the best sleep time ought to be ahead of time, we’ve fortunately observed that an overly high sleep time can have negligible effect on the total pull time and pull time can also continue to decrease even after reaching 100% success. Therefore, we recommend users lean towards a higher sleep time (e.g. 5.0 or 10.0 s for multiprocessing pulling) as a sleep time that’s too high has negligible effect while still obtaining 100% success, but a sleep time that’s too low can both increase the total pull time and lower the success percentage. Extra sleep time is needed when pulling only one entry at a time (e.g. greater than 5 s). We recommend that users take advantage of this ability of the KEGG API unless that option is not available for the entries they’d like to pull (i.e. Brite entries and entry fields that don’t support multiple entries within the response body). Considering the increase in success rate when pulling multiple entries per request as well as the significant decrease in pull time, it could be helpful for both users of kegg_pull and users of the KEGG API in general if KEGG both enabled support for pulling multiple entries for all entry and entry field types and even allowing more than 10 entries to be requested. All this applies to multi-processing, whereas the sleep time is not as important in single processing. As we’ve seen, even a sleep time of 0.0 can result in 100% success, likely because the time in between requests is already necessarily higher, preventing black listing.

**Table 11:** Feature comparison of packages with a similar purpose as kegg_pull

Package name	Can test requests before sending them	Both CLI and API included	Validates KEGG URLs	Wraps All KEGG API operations
kegg_pull	*Includes a test method in the API and –test option in the CLI for testing a URL without requesting it*	*Yes*	*If the URL is not valid, does not make the request and provides specific feedback*	*Provides wrapper methods and CLI commands for all the KEGG REST API operations*
KEGGutils	**No test method**	***API only***	*If the URL is not valid, does not make the request and provides specific feedback*	*Yes (some methods raise a file-not-found error)*
biopython (Bio.KEGG.REST)	**No test method**	***API only***	**Makes request without checking the URL first**	***Missing the “ddi” KEGG REST operation***
keggrest	**No test method**	***API only***	**Makes request without checking the URL first**	***Missing “ddi” and “info” KEGG REST operation***

Package Name	Makes a user-specified number of attempts per request	Pauses in between requests for a user-specified time to prevent blacklisting	Multiple output choices	User-specified timeout time
kegg_pull	*Tries making each request a number of times specified by the user, i.e. retries upon failure or time out*	*User specifies sleep time in between blacklisted requests to wait for access to the REST API to be restored*	*Pulled KEGG entries can either be saved in a regular directory or ZIP archive. Other output can be printed to the console, stored in a regular file, or stored in a ZIP file*	*User can specify how long a request can take until being considered timed out*
KEGGutils	**Attempts request once**	**Does not check for blacklisted requests**	**User does not choose output type**	**Time outs are not considered**
biopython (Bio.KEGG.REST)	**Attempts request once**	***Only allows 3 requests per second but user cannot specify wait time***	**N/A Does not save output as a file**	**Time outs are not considered**
keggrest	**Attempts request once**	**Does not check for blacklisted requests**	**User does not choose output type**	**Time outs are not considered**

Package Name	Separates entries and stores them individually	Saves binary KEGG entries in an appropriate format	Parses KEGG entry IDS
kegg_pull	*When making requests with multiple KEGG entries, separates and stores them in their individual files*	*Saves the binary image entries as binary files*	*For requests returning Entry IDs, parses them out such that only the entry IDs themselves are returned*
KEGGutils	**Can only pull one entry per request**	*Saves the image entries as image files (PNG OR GIF)*	**No command nor function for parsing entry IDs**
biopython (Bio.KEGG.REST)	**Neither saves nor separates entries**	**N/A Does not save entries**	**No command nor function for parsing entry IDs**
keggrest	**Can only pull one entry per request**	**Saves all output in the same format**	**No command nor function for parsing entry IDs**

Package Name	User can choose between multi-process and single process pulling	Can pull an arbitrary amount of entries	Specifies which entries succeeded, failed, or timed out
kegg_pull	*User can pull KEGG entries in a single process or a specified number of separate processes*	*Can pull an arbitrary number of user-specified entries with a single CLI or API command*	*When pulling KEGG entries, specifies by their entry ID which succeeded, failed, or timed out*
KEGGutils	**Single process only**	**Can only pull one entry in a function call**	**N/A only pulls one entry at a time**
biopython (Bio.KEGG.REST)	**Single process only**	**Can only pull as many entries as allowed in a KEGG REST “get” operation**	**Does not check for failure or time out of entries at all**
keggrest	**Single process only**	**Can only pull one entry in a function call**	**N/A only pulls one entry at a time**

Software feature descriptions highlighted in italic description fully reflect the feature, those highlighted in bold italic description partially reflect the feature, and those highlighted in bold description do not provide the feature at all

Since it’s still possible for entry requests to fail, we recommend users re-run kegg_pull on the failed entries after doing their best to initially select a good sleep time. This is not just because of blacklisting, but entries can inadvertently fail for other reasons such that they may succeed the second time. Entries that continuously fail to be pulled may be considered no longer available, as with the 20 consistently failed Brite entries. In such cases generally and in the case of the Brite database specifically, we recommend that KEGG either remove the IDs of these unavailable entries from the output of the “list” REST operation or that they troubleshoot to see whether these entries can be made available. We also recommend the—force-single-entry flag (CLI) or force_single_entry parameter (API) to be set if brite entry IDs are included in the call. While if a user chooses to pull the entire brite database, kegg_pull is smart enough to only pull one entry at a time. But it can’t know to do this if a file containing KEGG entries is provided.

Pulling KEGG entries into a ZIP archive is significantly slower both when multi-processing and when single processing. Likewise, single process pulling is significantly slower than multi-process pulling, both when pulling into a ZIP archive and when pulling into a regular directory. This means that multi-processing is still worth performing for ZIP archives despite locking multi-process unsafe code. Table 12 specifies the best decisions between multi-processing versus single processing and ZIP archives versus regular directories depending on the circumstances.

**Table 12:** Recommendation for multi-processing and storage options

Circumstance	Multiple cores available	Only one core available
Must store In ZIP archive	Multi-processing pull into a ZIP archive	Single processing pull into a ZIP Archive
No need For ZIP archive	Multi-processing pull into a regular directory	Single processing pull into a regular directory

The recommended manner of pulling based on the circumstance, with the need for a ZIP archive on the left side and the amount of cores available on the top

## Conclusions

The kegg_pull Python package provides the richest programmatic and command line access to the KEGG API to date. The clean object-oriented implementation provides robust multiprocessing KEGG entry retrieval (pull) functionality that is designed to mitigate blacklisting by the KEGG API. The kegg_pull API can be used in user-created Python scripts, while the CLI enables its use in data analysis pipelines and workflow managers, thus improving the FAIRness of KEGG. Furthermore, the CLI enables the creation of shell scripts that can fully document KEGG access for computational scientific reproducibility purposes. For users that prefer the command line, the CLI makes pulls from KEGG quick and easy, especially when organizing the pulled entries within a directory structure or utilizing other command line tools for search and analysis. The package is implemented to a high industrial software engineering standard, which includes both unit and integration tests that provides 100% code coverage. The code base is revision controlled and managed on GitHub, documentation is auto-updated onto associated GitHub Pages, and the package is distributed through the Python Package Index. Feedback is greatly appreciated. Any potential bugs or requests for new features can be submitted on our GitHub repository issues page here: https://github.com/MoseleyBioinformaticsLab/kegg_pull/issues.

## Supplementary Information

**Additional file 1**. Supplemental Material.

## Data Availability

GitHub repository: https://github.com/MoseleyBioinformaticsLab/kegg_pull. Python Package Index (PyPi): https://pypi.org/project/kegg-pull/. Documentation: https://moseleybioinformaticslab.github.io/kegg_pull/. Figshare containing this manuscript’s table results and the scripts to produce them: https://doi.org/10.6084/m9.figshare.21471990.

## Abbreviations

API: Application programming interface
CLI: Command line interface
KEGG: Kyoto Encyclopedia of Genes and Genomes
REST: Representational state transfer
URL: Uniform resource locator

## Availability and requirements

Project name: kegg_pull. Project home page: https://github.com/MoseleyBioinformaticsLab/kegg_pull. Operating system(s): Platform independent. Programming language: Python. Other requirements: Python3.8 or higher. License: Modified BSD 3 License. Report Bugs And Feature Requests Here: https://github.com/MoseleyBioinformaticsLab/kegg_pull/issues.
