# Supplementary material for: kegg_pull: a software package for the RESTful access and pulling from the Kyoto Encyclopedia of Gene and Genomes
Source: BMC Bioinformatics. 2023 Mar 4;24:78. doi: 10.1186/s12859-023-05208-0 (PMC9985241; doi:10.1186/s12859-023-05208-0)
Supplement: Supplementary file 1 — Additional file 1. Supplemental Material. [file 12859_2023_5208_MOESM1_ESM.docx]

**Supplementary Material for:**

kegg_pull: a Software Package for the RESTful Access and Pulling from The Kyoto Encyclopedia of Gene and Genomes

Erik Huckvale^1^ and Hunter N.B. Moseley^1,2,3,*^

^1^ Markey Cancer Center, University of Kentucky, Lexington, KY 40536, USA
^2^ Department of Molecular & Cellular Biochemistry, University of Kentucky, Lexington, KY 40536, USA
^3^ Institute for Biomedical Informatics, University of Kentucky, Lexington, KY 40536, USA
* corresponding author

[Additional Submodule Implementation Details 1](#_Toc127196811)

[kegg_url 1](#_Toc127196812)

[rest 2](#_Toc127196813)

[entry_ids 2](#_Toc127196814)

[pull 3](#_Toc127196815)

[Brite Failed Entries 3](#_Toc127196816)

[Single Process Pull Success Percentage And Time Spent Pulling By Sleep Time (KO Database) 4](#_Toc127196817)

# Additional Submodule Implementation Details

## kegg_url.py


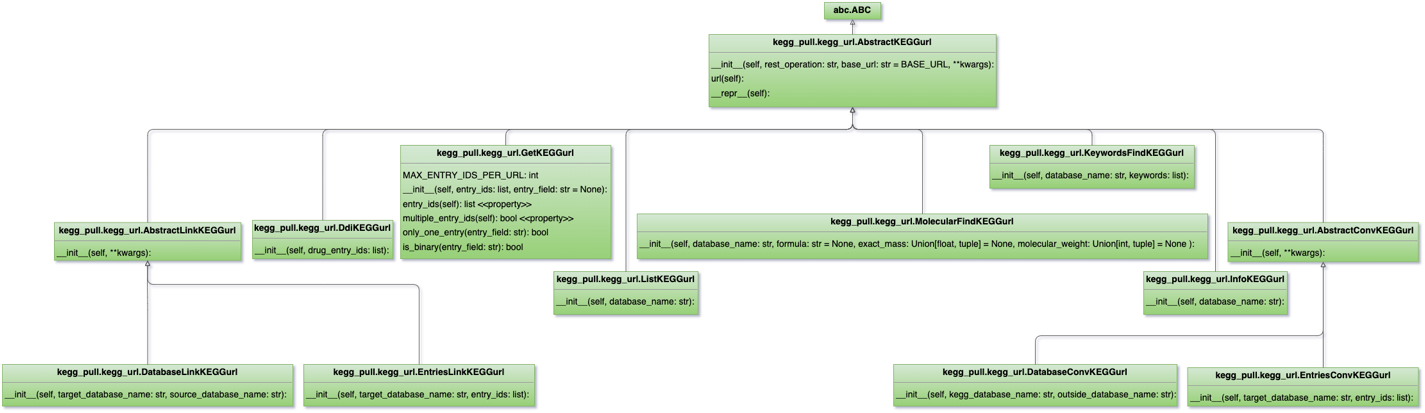


Figure S1 Class diagram of the kegg_url.py submodule.

Each URL class inherits from the AbstractKEGGurl class (Figure S1) with the shared “url” property, which is the constructed URL string itself. Each inheriting concrete class corresponds to one of the KEGG REST operations. Some of the KEGG REST operations have two forms, necessitating two different URL classes to represent both forms.

## rest.py


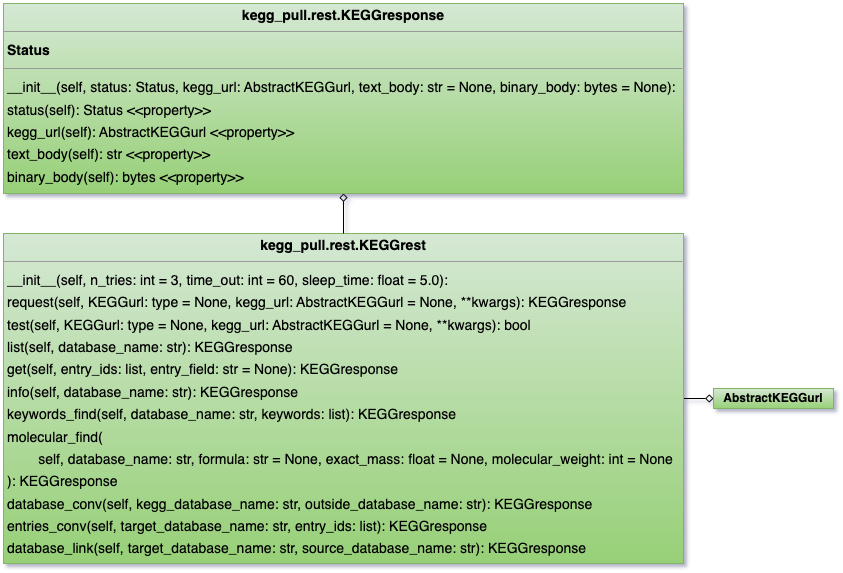


Figure S2 Class diagram of the rest.py submodule.

The rest submodule depends on the kegg_url submodule for providing wrapper methods over the KEGG REST API operations, contained within the KEGGrest class (Figure S2). Each wrapper method uses its corresponding KEGG URL class.

## entry_ids.py


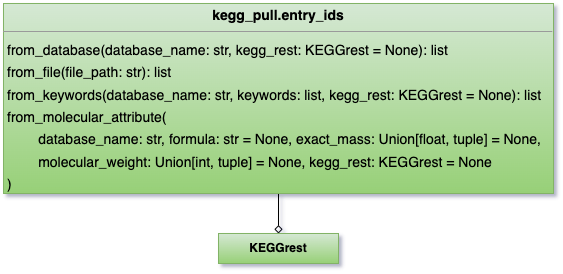


Figure S3 UML diagram of the entry_ids.py submodule.

The entry_ids submodule depends on the rest submodule, since it uses the KEGGrest class’s methods for obtaining entry IDs (i.e. the “list” method for getting all the entry IDs of a given KEGG database and the keywords_find and molecular_find methods for getting the entry IDs using the “find” KEGG REST operation). These methods accept an optional KEGGrest object, allowing the user to customize how requests are made if desired (e.g. the time to wait for a time out, etc.) except for the from_file method (Figure S3).

## pull.py

The pull submodule depends on the rest submodule for obtaining KEGG entries given their IDs. Within the submodule is the SinglePull class which contains a KEGGrest object (see Figure 2) to use its “get” method followed by extracting one or more KEGG entries from the text body (string) of the KEGGresponse returned or the binary body (bytes) in the case of “image” fields from relevant entries. While “image” can be set as the entry_field parameter to the pull method, all the other entry fields for the “get” KEGG REST operation can also be set with this parameter to pull particular fields from entries. If unspecified, the entry itself will be pulled in its default format. The SinglePull class performs just one request in its pull method, as its name suggests. Currently, there is a limit of 10 entry IDs that can be sent in this request, since KEGG API’s response will truncate to 10 entries if more are requested. So, the SinglePull class is limited on how many entries it can pull for a single command.

The SingleProcessMultiplePull and MultiProcessMultiplePull (see Figure 2) on the other hand are not limited to a maximum number of entries and can pull an arbitary amount. These contain a SinglePull object and call its “pull” method as many times as necessary to request all the entry IDs provided to their own “pull” method. As a result, there is no limit to the number of entry IDs that can be provided, unlike with the SinglePull class. The “pull” method of these classes, like that of the SinglePull class, also returns a PullResult object detailing which of the requested entry IDs succeeded, failed, or timed out. They merge the PullResult objects returned by each individual call of their SinglePull member’s “pull” method into a comprehensive PullResult object.

Since writing to a ZIP archive is not multi-processing safe, the MultiProcessMultiplePull necessitates that its SinglePull member (Figure 2) have its multi_process_lock_save parameter set to True when its output parameter is a ZIP archive. This results in the SinglePull using a Lock object (from the built-in multiprocessing Python library) to process lock the code block that writes to the ZIP archive, meaning while one process opens and writes to the ZIP archive, other processes are temporarily blocked from doing so. This means a slight reduction in performance since the other processes are delayed whenever they happen to come to the ZIP archive writing code at the same time as another process, but the majority of the code executed across multiple processes is not locked, resulting in an increase in efficiency even when writing to a ZIP archive file (See table 5).

# Brite Failed Entries

br:br03220

br:br03222

br:br01610

br:br01611

br:br01612

br:br01613

br:br01601

br:br01602

br:br01600

br:br01620

br:br01553

br:br01554

br:br01556

br:br01555

br:br01557

br:br01800

br:br01810

br:br08011

br:br08020

br:br08120

# Single Process Pull Success Percentage And Time Spent Pulling By Sleep Time (KO Database)

| **Sleep Time (seconds)** | **0.0** | **0.5** | **1.0** | **2.0** | **3.0** | **5.0** | **10.0** |
| --- | --- | --- | --- | --- | --- | --- | --- |
| **Percent Success** | 100.0 | 100.0 | 100.0 | 100.0 | 100.0 | 100.0 | 100.0 |
| **Pull Time (minutes)** | 70.88 | 72.48 | 72.27 | 71.95 | 72.36 | 72.37 | 72.36 |
